# Utilization Rates of Pancreatectomy, Radical Prostatectomy, and Nephrectomy in New York, Ontario, and New South Wales, 2011 to 2018

**DOI:** 10.1001/jamanetworkopen.2021.5477

**Published:** 2021-04-19

**Authors:** Hilary Y. M. Pang, Kelsey Chalmers, Bruce Landon, Adam G. Elshaug, John Matelski, Vicki Ling, Monika K. Krzyzanowska, Girish Kulkarni, Bradley A. Erickson, Peter Cram

**Affiliations:** 1 Temerty Faculty of Medicine, University of Toronto, Toronto, Ontario, Canada; 2 Institute of Health Policy, Management, and Evaluation, Dalla Lana School of Public Health, University of Toronto, Toronto, Ontario, Canada; 3 Menzies Centre for Health Policy, School of Public Health, University of Sydney, Sydney, New South Wales, Australia; 4 Lown Institute, Brookline, Massachusetts; 5 Department of Health Care Policy, Harvard Medical School, Boston, Massachusetts; 6 Division of General Medicine, Beth Israel Deaconess Medical Center, Boston, Massachusetts; 7 Centre for Health Policy, Melbourne School of Population and Global Health and the Melbourne Medical School, University of Melbourne, Melbourne, Victoria, Australia; 8 USC–Brookings Schaeffer Initiative for Health Policy, The Brookings Institution, Washington, DC; 9 Biostatistics Research Unit, Toronto General Hospital, Toronto, Ontario, Canada; 10 ICES Sciences, Toronto, Ontario, Canada; 11 Department of Medical Oncology and Haematology, Princess Margaret Cancer Centre, University Health Network, Toronto, Ontario, Canada; 12 Department of Surgical Oncology, Princess Margaret Cancer Centre, University Health Network, Toronto, Ontario, Canada; 13 Department of Urology, University of Iowa and Clinics, Iowa City; 14 Department of General Internal Medicine, University Health Network and Sinai Health Systems, Toronto, Ontario, Canada

## Abstract

**Importance:**

Few studies have compared surgical utilization between countries or how rates may differ according to patients’ socioeconomic status.

**Objective:**

To compare population-level utilization of 3 common nonemergent surgical procedures in New York State (US), Ontario (Canada), and New South Wales (Australia) and how utilization differs for residents of lower- and higher-income neighborhoods.

**Design, Setting, and Participants:**

This cohort study included all adults aged 18 years and older who were hospitalized for pancreatectomy, radical prostatectomy, or nephrectomy between 2011 and 2016 in New York, between 2011 and 2018 in Ontario, and between 2013 and 2018 in New South Wales. Each patient’s address of residence was linked to 2016 census data to ascertain neighborhood income. Data were analyzed from August 2019 to November 2020.

**Main Outcomes and Measures:**

Primary outcomes were (1) each jurisdiction’s per capita age- and sex-standardized utilization rates (procedures per 100 000 residents per year) for each surgery and (2) utilization rates among residents of lower- and higher-income neighborhoods.

**Results:**

This study included 115 428 surgical patients (25 780 [22.3%] women); 5717, 21 752, and 24 617 patients in New York were hospitalized for pancreatectomy, radical prostatectomy, and nephrectomy, respectively; 4929, 19 125, and 16 916 patients in Ontario, respectively; and 2069, 13 499, and 6804 patients in New South Wales, respectively. Patients in New South Wales were older for all procedures (eg, radical prostatectomy, mean [SD] age in New South Wales, 64.8 [7.3] years; in New York, 62.7 [8.4] years; in Ontario, 62.8 [6.7] years; *P* < .001); patients in New York were more likely than those in other locations to be women for pancreatectomy (New York: 2926 [51.2%]; Ontario: 2372 [48.1%]; New South Wales, 1003 [48.5%]; *P* = .004) and nephrectomy (New York: 10 645 [43.2%]; Ontario: 6529 [38.6%]; 2605 [38.3%]; *P* < .001). With the exception of nephrectomy in Ontario, there was a higher annual utilization rate for all procedures in all jurisdictions among patients residing in affluent neighborhoods (quintile 5) compared with poorer neighborhoods (quintile 1). This difference was largest in New South Wales for pancreatectomy (4.65 additional procedures per 100 000 residents [SE, 0.28]; *P* < .001) and radical prostatectomy (73.46 additional procedures per 100 000 residents [SE, 1.20]; *P* < .001); largest in New York for nephrectomy (8.43 additional procedures per 100 000 residents [SE, 0.85]; *P* < .001) and smallest in New York for radical prostatectomy (19.70 additional procedures per 100 000 residents [SE, 2.63]; *P* < .001); and smallest in Ontario for pancreatectomy (1.15 additional procedures per 100 000 residents [SE, 0.28]; *P* < .001) and nephrectomy (−1.10 additional procedures per 100 000 residents [SE, 0.52]; *P* < .001). New York had the highest utilization of nephrectomy (28.93 procedures per 100 000 residents per year [SE, 0.18]) and New South Wales for had the highest utilization of pancreatectomy and radical prostatectomy (6.94 procedures per 100 000 residents per year [SE, 0.15] and 94.37 procedures per 100 000 residents per year [SE, 0.81], respectively; all *P* < .001). Utilization was lowest in Ontario for all procedures (pancreatectomy, 6.18 procedures per 100 000 residents per year [SE, 0.09]; radical prostatectomy, 49.24 procedures per 100 000 residents per year [SE, 0.36]; nephrectomy, 21.40 procedures per 100 000 residents per year [SE, 0.16]; all *P* < .001).

**Conclusions and Relevance:**

In this study, New York and New South Wales had higher per capita surgical utilization and larger neighborhood income–utilization gradients than Ontario. These findings suggest that income-based disparities are larger in the United States and Australia and smaller in Canada and highlight trade-offs inherent in the health care systems of different countries.

## Key Points

**Question:**

Do utilization rates for pancreatectomy, radical prostatectomy, and nephrectomy differ between New York State (US), Ontario (Canada), and New South Wales (Australia), and do income-based differences in utilization vary between countries?

**Findings:**

This cohort study of 115 428 surgical patients found significantly lower surgical utilization in Ontario than in New York and New South Wales. Residents of lower-income neighborhoods had lower rates of surgery than residents of higher-income neighborhoods in all countries; income-based differences were significantly smaller in Ontario than in New York and New South Wales.

**Meaning:**

In this study, Ontario had lower surgical utilization rates and smaller differences in utilization between patients in high-income vs low-income neighborhoods, but income-based disparities were present in all jurisdictions.

## Teaser

This cohort study compares population-level utilization of pancreatectomy, radical prostatectomy, and nephrectomy in New York (US), Ontario (Canada), and New South Wales (Australia) and how utilization differs for residents of lower- and higher-income neighborhoods.

## Introduction

The US health care system is recognized for world-class tertiary and quaternary care for those who are able to pay but poorer outcomes and limited access for those who cannot.^1,2^ In the US, there is also growing concern about overuse of certain surgical procedures, motivated by easy access to imaging and diagnostic testing.^3,4,5,6^ Interestingly, there are very few population-based studies comparing surgical utilization rates between countries.^7,8^ Moreover, few studies have examined whether the strong positive association between surgical utilization rates and socioeconomic status (SES) that have been documented in the US also exist in other countries where health insurance coverage differs.

In contrast to the US, both Canada and Australia have government-sponsored insurance programs that cover all legal residents. Canada generally prohibits private insurance and is thought to have more equitable access to services than the US, but many have raised concerns over inadequate access to many medical services.^9,10^ The Australian public system similarly has long queues, but approximately 50% of the population purchases supplemental private insurance that allows for improved access, particularly for surgical services.^11,12,13^ Few studies have directly compared utilization of health services in the US, Canada, and Australia, and whether differences in utilization across strata of SES might vary between countries.

We used population-based administrative data from New York State (US), Ontario (Canada), and New South Wales (Australia) to compare utilization rates of the following 3 surgical procedures: pancreatectomy, radical prostatectomy, and nephrectomy. We selected these procedures because they are common, usually performed in the inpatient setting, and would have major public health impacts if underused (ie, patients not receiving needed surgery) or overused (ie, unnecessary morbidity and mortality). Moreover, the juxtaposition of these procedures provides an interesting contrast between jurisdictions; pancreatectomy and nephrectomy have few nonsurgical alternatives, while radical prostatectomy has several. Thus, these 3 procedures offer a rich perspective on care delivery across the 3 countries with dissimilar health care financing programs. Our primary hypotheses were that utilization of all procedures would be significantly higher in New York relative to Ontario and New South Wales and that differences in utilization between patients of lower and higher SES would be significantly greater in New York and smaller in Ontario.

## Methods

This retrospective cohort study used administrative data from New York (2016 population, 19.7 million), Ontario (2016 population, 13.4 million) and New South Wales (2016 population, 7.7 million) to identify all hospitalized adults in each jurisdiction who underwent pancreatectomy, radical prostatectomy, and nephrectomy. We chose these 3 Organization for Economic Co-operation and Development (OECD) countries for comparison because of their racial, ethnic, and geographic diversity, large populations, and close geopolitical ties but vastly different health care systems.

This study received ethical approval by the research ethics boards at ICES, University Health Network, and the New South Wales Population and Health Services Research Ethics Committee. A waiver of informed consent was granted because the population-based databases used in this study were large, and obtaining informed consent was considered not feasible for this study. This study followed the reporting requirements of the Strengthening the Reporting of Observational Studies in Epidemiology (STROBE) reporting guideline.

### Data Sources

For New York, we used data from the 2011 to 2016 State Inpatient Database (SID), which has been used extensively in prior research.^14^ The SID contains administrative data for all patients admitted to all nongovernmental acute care hospitals in the state, including individuals covered by Medicare, Medicaid, or private insurance as well as those who are uninsured. Data elements for each admission include patient demographic characteristics (including zip code of residence), primary and secondary diagnosis and procedures using *International Classification of Diseases, Ninth Revision *(*ICD-9*) and *ICD-10* codes, and discharge disposition (eg, home, died). The SID also assigns each patient a unique identifier that allows patients to be tracked for purposes of interhospital transfer or hospital readmission. The SID does not capture out-of-hospital care or deaths.

For Ontario, we used the 2011 to 2018 Discharge Abstract Database (DAD), which provides information on all hospitalizations paid for by the Ontario Health Insurance Plan (OHIP); OHIP provides health insurance to all legal residents of Ontario (approximately 99% of the population) and 100% of inpatient hospitalizations.^15^ Ontario’s DAD contains analogous data elements to the SID, including demographic characteristics, primary and secondary diagnoses using *ICD-10 Canadian modification* (*ICD-10-CA*) codes, and procedures captured using Canadian Classification of Health Interventions (CCI) codes.

For New South Wales, we used the 2013 to 2018 Admitted Patient Data Collection (APDC), which records all inpatient hospitalizations within the state and is often used for health services research.^16^ Key data elements for each hospitalization include demographic characteristics, a unique facility identifier, unique patient identifier, and discharge disposition. Procedures are recorded using the Australian Classification of Health Interventions and diagnosis codes are recorded using *ICD-10 Australian Modification* codes (*ICD-10-AM*). The SID, DAD, and APDC each contain nearly 100% of hospital inpatient claims for New York State, Ontario, and New South Wales, respectively.

### Cohort Generation

We identified all patients aged 18 to 105 years who underwent initial pancreatectomy, radical prostatectomy, or nephrectomy between January 1, 2011, (Ontario and New York) or January 1, 2013, (New South Wales) and March 31, 2018, (Ontario and New South Wales) or September 30, 2016, (New York) excluding those who had received the same procedure during a 90-day look-back period to avoid counting readmissions as de novo procedures (eFigure 1 in the Supplement). Differing timelines reflected differences in data availability and access across jurisdictions. Patients were identified using relevant *ICD-9-CM*, *ICD-10*, CCI, or *ICD-10-AM* codes listed in the primary procedure field using published algorithms in conjunction with local content experts (eAppendix in the Supplement). Our full study protocol and hypotheses were developed a priori and are available on Open Science Framework.^17^

We excluded patients discharged after September 30, 2016, (New York) and March 31, 2018, (Ontario and New South Wales) to allow for 90 days of postdischarge follow-up (eFigure 1 in the Supplement). We excluded patients who had missing age, sex, or hospital identification number, resided outside the jurisdiction where they received their surgery, had hospital length of stay (LOS) of less than 1 day, or received their procedures in hospitals that performed less than 1 surgery per year. We created a single linked episode of care for patients who underwent interhospital transfers.

We linked the address of residence for each patient to publicly available 2016 census data available for each jurisdiction (US Census Bureau, Statistics Canada, and the Australian Bureau of Statistics) to ascertain neighborhood income.^18,19,20^ We then stratified the neighborhoods in each jurisdiction into income quintiles (with quintile 1 indicating lowest income and quintile 5 indicating highest income).

### Statistical Analysis

First, we compared the demographic characteristics of patients hospitalized for pancreatectomy, radical prostatectomy, and nephrectomy across New York State, Ontario, and New South Wales using bivariate methods. We used similar methods to compare the proportion of patients residing in higher- and lower-income neighborhoods and the proportion of patients who underwent interhospital transfers.

Second, we compared the proportion of acute care hospitals in each jurisdiction that performed each surgery as well as annual surgical volumes. Importantly, our comparisons of patient characteristics and hospital characteristics were secondary analyses and should be considered as such.

Third, to evaluate our primary hypotheses, we used direct standardization (Ontario population reference) to compare per capita rates of pancreatectomy, radical prostatectomy, and nephrectomy (procedures per 100 000 residents per year) between jurisdictions.^21^ The numerator was the total number of procedures performed in each jurisdiction; the denominator was the number of eligible adults in that jurisdiction (only men for radical prostatectomy) with rates annualized to account for study period differences. We then compared utilization rates across jurisdictions after stratifying by neighborhood income quintile. These comparisons constituted our primary outcomes.

Fourth, we conducted stratified analyses comparing utilization across jurisdictions in subgroups defined by decade of age and sex. Finally, we compared short-term outcomes for each surgery between our 3 jurisdictions using generalized estimating equations. Outcomes included: hospital LOS (excluding LOS >100 days); discharge disposition (death, home, nonhome care facility [ie, long-term care, nursing home, or rehabilitation center], or other); and hospital readmission within 30 days and 90 days of discharge among those who survived to discharge. We examined both overall in-hospital mortality and in-hospital mortality within 7 days of surgery. We compared unadjusted outcomes, outcomes standardized for age- and sex-differences between jurisdictions (model 1), and outcomes standardized by all model 1 factors plus hospital volume and neighborhood income (model 2). We did not standardize for differences in comorbid illness because of implausibly large differences in coding demonstrated previously.^8,22^

Statistical significance was set at *P* < .05 a priori, and all tests were 2-sided. Analyses were performed using SAS version 9.4 (SAS Institute) or R statistical software version 3.6.3 (R Project for Statistical Computing) packages. We did not make any adjustment for multiple comparisons.

## Results

This study included 115 428 surgical patients (25 780 [22.3%] women); 5717, 21 752, and 24 617 patients in New York hospitalized for pancreatectomy, radical prostatectomy, and nephrectomy, respectively; 4929, 19 125, and 16 916 patients in Ontario, respectively; and 2069, 13 499, and 6804 patients in New South Wales, respectively (Table 1). For pancreatectomy and nephrectomy, patients in New York were more likely to be women compared with Ontario and New South Wales (pancreatectomy: New York: 2926 [51.2%]; Ontario: 2372 [48.1%]; New South Wales, 1003 [48.5%]; *P* = .004; nephrectomy: New York: 10 645 [43.2%]; Ontario: 6529 [38.6%]; 2605 [38.3%]; *P* < .001). Patients in New South Wales were slightly older for all 3 procedures (eg, radical prostatectomy, mean [SD] age in New South Wales, 64.8 [7.3] years; in New York, 62.7 [8.4] years; in Ontario, 62.8 [6.7] years; *P* < .001). Patients in New York had a significantly higher prevalence of most comorbid conditions (eTable 1 in the Supplement).

**Table 1.** Patient and Hospital Characteristics and Per-Capita Standardized Utilization Rates for Pancreatectomy, Radical Prostatectomy, and Nephrectomy for Recipients From New York (US), Ontario (Canada), and New South Wales (Australia)

Characteristic	Pancreatectomy				Radical Prostatectomy				Nephrectomy			
	No. (%)			*P* value	No. (%)			*P* value	No. (%)			*P* value
	New York State	Ontario	New South Wales		New York State	Ontario	New South Wales		New York State	Ontario	New South Wales	
**Patient characteristics**												
No.	5717	4929	2069	NA	21 752	19 125	13 499	NA	24 617	16 916	6804	NA
Age, mean (SD), y	63.1 (13.4)	62.5 (13.4)	64.3 (13.0)	<.001	62.7 (8.4)	62.8 (6.7)	64.8 (7.3)	<.001	59.1 (14.9)	61.7 (13.4)	63.0 (13.4)	<.001
Women	2926 (51.2)	2372 (48.1)	1003 (48.5)	.004	NA	NA	NA	NA	10 645 (43.2)	6529 (38.6)	2605 (38.3)	<.001
Men	2791 (48.8)	2557 (51.9)	1066 (51.5)		21 752 (100)	19 125 (100)	13 499 (100)		13 972 (56.8)	10 387 (61.4)	4199 (61.7)	
Neighborhood income quintile, No. (%)												
1, lowest 20%	1062 (18.6)	868 (17.6)	2670 (13.1)	<.001	4098 (18.8)	2573 (10.9)	1760 (13.1)	.004	4833 (19.6)	3261 (19.3)	956 (14.1)	<.001
2	766 (13.4)	996 (20.2)	347 (16.8)	<.001	2893 (13.3)	3493 (18.3)	2353 (17.4)	<.001	3538 (14.4)	3414 (20.2)	1364 (20.1)	<.001
3	852 (14.9)	969 (19.7)	408 (19.7)	<.001	3530 (16.2)	3783 (19.8)	2412 (17.9)	<.001	4135 (16.8)	3333 (19.7)	1428 (21.0)	<.001
4	1245 (21.8)	965 (19.6)	479 (23.1)	<.001	4631 (21.3)	4210 (22.0)	3273 (24.3)	.006	5055 (20.5)	3512 (20.8)	1623 (23.9)	<.001
5, highest 20%	1651 (28.9)	1118 (22.7)	566 (27.3)	<.001	6143 (28.2)	5028 (26.3)	3697 (27.4)	<.001	6513 (26.5)	3359 (19.9)	1432 (21.1)	<.001
Missing	141 (2.5)	13 (0.3)	0		457 (2.1)	38 (0.2)	0		543 (2.2)	37 (0.2)	SC^a^	
Difference, quintile 5–quintile 1	589 (10.3)	250 (5.1)	295.7 (14.3)	<.001	2045 (9.4)	2455 (12.8)	1937 (14.3)	<.001	1680 (6.8)	98 (0.6)	476 (7.0)	<.001
Underwent interhospital transfer	20 (0.3)	140 (2.8)	202 (9.7)	<.001	18 (0.1)	27 (0.1)	159 (1.2)	<.001	46 (0.2)	175 (1.0)	212 (3.1)	<.001
**Hospital characteristics**												
No.	224	166	314	NA	224	166	314	NA	224	166	314	NA
Hospitals performing surgery	55 (24.6)	23 (13.9)	42 (13.4)	.001	123 (54.9)	56 (33.7)	62 (19.8)	<.001	126 (56.2)	60 (36.1)	61 (19.4)	<.001
Annual surgical volume, mean (SD), No.	18.2 (29.0)	29.7 (41.6)	11.2 (12.1)	.04	30.8 (52.1)	47.2 (55.3)	41.0 (61.4)	.20	34.5 (55.2)	39.4 (43.6)	21.3 (19.1)	.08
Annual surgical volume, median (IQR)	8 (2-21)	7 (2-39)	7 (2-20)	NA	9 (3-33)	33 (17-55)	19 (9-37)	NA	12 (4-32)	27 (13-47)	16 (6-32)	NA
**Utilization rate, Procedures per 100 000 residents (SE)**												
Overall per capita rate	6.68 (0.09)	6.18 (0.09)	6.94 (0.15)	<.001	54.01 (0.37)	49.24 (0.36)	94.37 (0.81)	<.001	28.93 (0.18)	21.40 (0.16)	23.03 (0.28)	<.001
Sex												
Men	6.96 (0.13)	6.61 (0.13)	7.33 (0.23)	.01	54.01 (0.37)	49.24 (0.36)	94.37 (0.81)	<.001	34.88 (0.29)	27.17 (0.26)	29.27 (0.45)	<.001
Women	6.41 (0.12)	5.77 (0.12)	6.58 (0.21)	<.001	NA	NA	NA	NA	23.35 (0.23)	15.98 (0.20)	17.17 (0.34)	<.001
Difference, male-female, (SE)	0.56 (0.18)	0.83 (0.18)	0.74 (0.31)	.57	NA	NA	NA	NA	11.53 (0.37)	11.19 (0.33)	12.10 (0.56)	.37
*P* value by region	.002	<.001	.02	NA	NA	NA	NA	NA	<.001	<.001	<.001	NA

Abbreviations: IQR, interquartile range; NA, not applicable; SC, suppressed cell.

^a^ Cell sizes of less than 5 (Ontario and New South Wales) or less than 10 (New York) were suppressed in accordance with jurisdictional research ethics board policy.

Hospitals in New York were significantly more likely to perform each of the 3 procedures than those in Ontario and New South Wales. However, there were more low volume hospitals in New York and New South Wales relative to Ontario, as assessed by interquartile range (Table 1).

Focusing on our primary outcomes, New York had the highest utilization for nephrectomy (28.93 procedures per 100 000 residents per year [SE, 0.18]; *P* < .001), while New South Wales had the highest utilization of pancreatectomy (6.94 procedures per 100 000 residents per year [SE, 0.15]; *P* < .001) and radical prostatectomy (94.37 procedures per 100 000 residents per year [SE, 0.81]; *P* < .001) (Table 1). Ontario had the lowest utilization for all 3 procedures (pancreatectomy, 6.18 procedures per 100 000 residents per year [SE, 0.09]; radical prostatectomy, 49.24 procedures per 100 000 residents per year [SE, 0.36]; nephrectomy, 21.40 procedures per 100 000 residents per year [SE, 0.16]; all *P* < .001) (Table 1).

**Figure 1.**
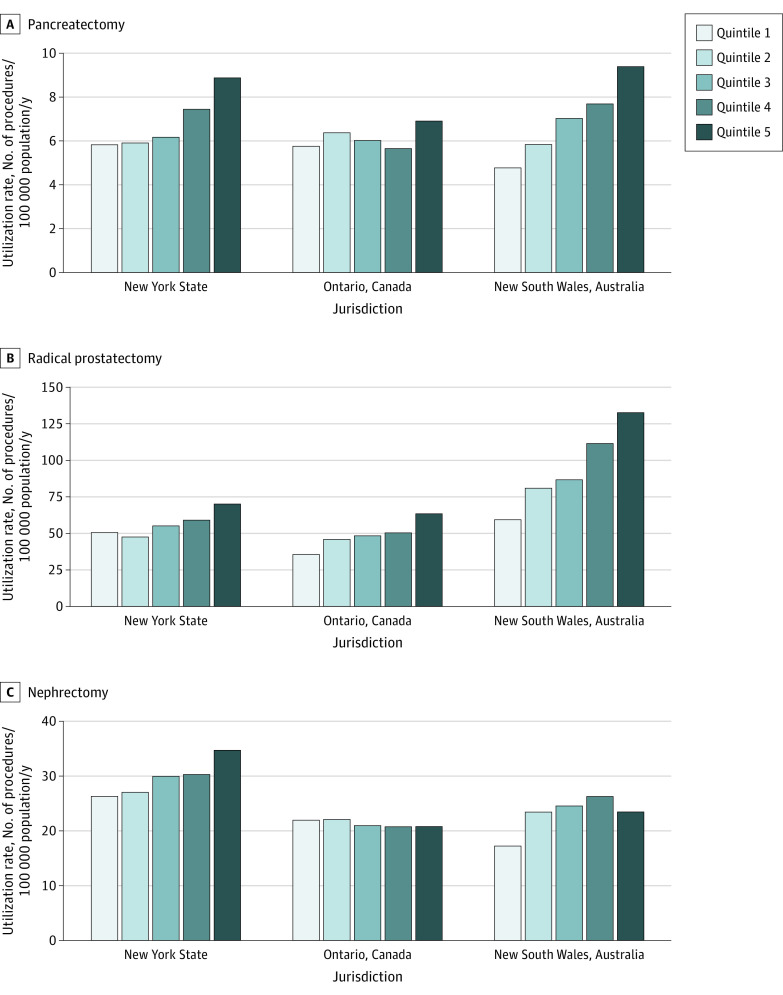
Standardized Per Capita Utilization of Pancreatectomy, Radical Prostatectomy, and Nephrectomy by Neighborhood Income Quintile in New York State, Ontario, and New South Wales Quintile 1 indicates lowest income neighborhood; quintile 5, highest income neighborhood.

Utilization rates were significantly higher for residents of the highest income neighborhoods (quintile 5) compared with the lowest income neighborhoods (quintile 1) for all procedures in all jurisdictions, with the exception of nephrectomy in Ontario (Table 2 and Figure 1). Viewed through a different lens, New South Wales had the largest difference in utilization between quintile 5 and quintile 1 for both pancreatectomy (4.65 additional procedures per 100 000 residents; SE, 0.28) and radical prostatectomy (difference, 73.46 procedures per 100 000 residents; SE, 1.20), while New York had the largest difference for nephrectomy (difference, 8.43 procedures per 100 000 residents; SE, 0.85) but the smallest difference for radical prostatectomy (difference, 19.70 procedures per 100 000 residents; SE, 2.63). Ontario had the smallest gradient for both pancreatectomy (difference, 1.15 procedures per 100 000 residents; SE, 0.28) and nephrectomy (difference, −1.10 procedures per 100 000 residents; SE, 0.52).

**Table 2.** Standardized Utilization Rate by Neighborhood Income Quintile for Pancreatectomy, Radical Prostatectomy, and Nephrectomy for Recipients From New York, Ontario, and New South Wales

Neighborhood income quintile	Pancreatectomy				Radical Prostatectomy				Nephrectomy			
	Procedures/100 000 residents, No.			*P* value	Procedures/100 000 residents, No.			*P* value	Procedures/100 000 residents, No.			*P* value
	NY	ON	NSW		NY	ON	NSW		NY	ON	NSW	
1, lowest 20%	5.83	5.78	4.77	.001	50.56	35.57	59.55	<.001	26.35	21.97	17.29	<.001
2	5.92	6.38	5.85	.20	47.53	46.00	81.23	<.001	27.06	22.10	23.49	<.001
3	6.18	6.04	7.04	.04	55.09	48.5	87.16	<.001	30.06	21.03	24.68	<.001
4	7.46	5.67	7.70	<.001	59.23	50.75	111.85	<.001	30.41	20.88	26.30	<.001
5, highest 20%	8.88	6.93	9.42	<.001	70.26	63.51	133.00	<.001	34.78	20.87	23.52	<.001
*P* value	<.001	<.001	<.001	NA	<.001	<.001	<.001	NA	<.001	<.001	0.036	NA
5 vs 1, difference (SE)	3.05 (0.49)	1.15 (0.28)	4.65 (0.28)	<.001	19.70 (2.63)	27.94 (1.14)	73.46 (1.20)	<.001	8.43 (0.85)	−1.10 (0.52)	6.23 (0.57)	<.001
5 to 1 ratio	1.52	1.20	1.97		1.39	1.79	2.23		1.32	0.95	1.36	
Regional comparison	NY v ON	NSW vs ON	NSW vs NY	NA	NY vs ON	NSW vs ON	NSW vs NY	NA	NY vs ON	NSW vs ON	NSW vs NY	NA
Quintile 5 vs 1 *P* value	.001	<.001	.005	NA	.004	<.001	<.001	NA	<.001	<.001	.031	NA

Abbreviations: NA, not applicable; NSW, New South Wales; NY, New York; ON, Ontario.

**Figure 2.**
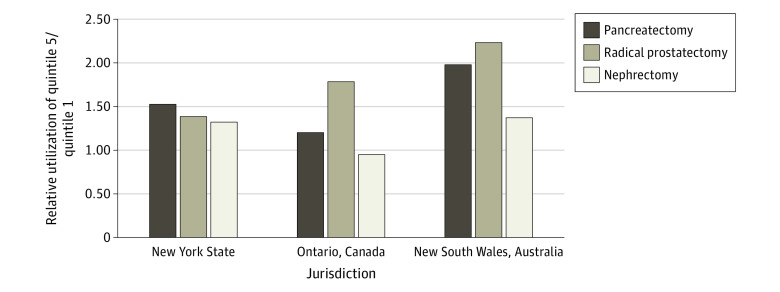
Relative Utilization of Pancreatectomy, Radical Prostatectomy, and Nephrectomy in Neighborhood Income Quintile 5 vs Quintile 1 in New York State, Ontario, and New South Wales A relative utilization of greater than 1.00 indicates higher utilization for residents in the highest income neighborhoods (quintile 5) relative to the lowest income neighborhoods (quintile 1). A relative utilization of less than 1.00 indicates lower utilization for residents in the highest income neighborhoods (quintile 5) relative to the lowest income neighborhoods (quintile 1).

Looking instead at the relative utilization for residents of neighborhood income quintile 5 vs quintile 1 (Table 2 and Figure 2), residents of wealthy neighborhoods in New York had relatively similar increases in utilization for all 3 procedures compared with residents of poorer neighborhoods (ratio of quintile 5 to 1: pancreatectomy, 1.52; radical prostatectomy, 1.39; nephrectomy, 1.32). In contrast, the neighborhood income–based differences were highly variable in both New South Wales (ratio of quintile 5 to 1: pancreatectomy, 1.97; radical prostatectomy, 2.23; nephrectomy, 1.36) and Ontario (ratio of quintile 5 to 1: pancreatectomy, 1.20; radical prostatectomy, 1.79; nephrectomy, 0.95) (Figure 2).

In subgroup analyses, we found that utilization of pancreatectomy and nephrectomy was significantly higher for men than women in all jurisdictions (Table 1). Utilization of all 3 procedures increased until age 70 to 79 years, before declining at age 80 years and older (eFigure 2 in the Supplement).

Secondary outcomes demonstrated that in-hospital mortality was significantly higher in Ontario for pancreatectomy and nephrectomy in unadjusted analyses, although differences were attenuated in risk-standardized analysis (eTable 2 and eTable 3 in the Supplement). Rates of readmission within 30 days and 90 days of surgery were generally similar between jurisdictions (eTable 2 and eTable 3 in the Supplement).

## Discussion

In an analysis of population-based administrative data from New York State (US), Ontario (Canada), and New South Wales (Australia), we found higher overall utilization rates of pancreatectomy, radical prostatectomy, and nephrectomy in the US and Australia compared with Canada for all procedures. Residents of higher-income neighborhoods in all 3 jurisdictions were significantly more likely to undergo all 3 surgical procedures compared with residents of lower-income neighborhoods. However, the neighborhood income–utilization gradient was larger in New York State and New South Wales than in Ontario. In aggregate, our results suggest that while all jurisdictions have some degree of income-based disparities, the magnitude of these differences is smaller in Canada and larger in the US and Australia. Our findings highlight how countries’ health care systems can exacerbate or alleviate wealth-based differences in access to surgical procedures.

Several aspects of our study warrant elaboration. First, it is important to consider the overall differences in utilization rates between jurisdictions. Ontario had the lowest per capita utilization rate for all 3 procedures (8%, 9%, and 26% lower than New York for pancreatectomy, radical prostatectomy, and nephrectomy, respectively). There have been longstanding concerns in Canada regarding wait times for surgery but few direct comparisons with other countries.^23,24^ While the debate over access is often highly politicized, our results expand on evidence of markedly lower surgical utilization rates in Canada relative to not only to the US but also to other high-income countries.^8,14,22,25^

When considering the overall utilization rates of these 3 procedures, it is important to recognize that each surgery is predominantly performed for the treatment of cancer. While it may be tempting to conclude that the differences in surgical utilization between countries reflect underlying differences in cancer incidence, this seems biologically implausible.^26,27,28^ A more likely explanation is that the differences in surgical utilization reflect a complex diagnostic cascade that begins with between-country differences in cancer screening practices (such as prostate specific antigen [PSA] testing) and extending to differences in the supply of surgeons and hospital operating room capacity.^3,29,30,31,32,33,34,35^ Our finding that New South Wales had markedly higher rates of radical prostatectomy compared with New York and Ontario is consistent with similar single-jurisdiction studies.^36,37,38^ The US and Canada had more conservative PSA screening guidelines than Australia during the study period; US and Canadian guidelines did not recommend PSA testing for prostate cancer screening in healthy asymptomatic men, while Australia supported testing if requested and if an informed discussion was had on the benefits and harms of testing.^39,40,41^ Further studies are needed to better understand the drivers of the differences in utilization across jurisdictions we have identified.

It is also important to recognize that jurisdictions may have differences in terms of preferences for surgical and nonsurgical treatment for prostate cancer. Pancreatectomy and nephrectomy constitute the principal treatments for resectable pancreatic and kidney cancer, respectively, whereas localized prostate cancer can be managed with watchful waiting, radiation, or surgery.^42,43,44,45^ Further research is needed to elucidate whether the higher rate of radical prostatectomy in New South Wales could be counterbalanced by higher utilization of nonsurgical management (ie, radiation, watchful waiting) in New York and Ontario.

Second, our finding of higher utilization of all 3 procedures for residents of higher-income neighborhoods in all 3 countries extends prior research linking higher-income and increased health care access.^2,46,47,48^ Single-country studies have demonstrated that wealthier patients are likelier to obtain cancer screening, undergo cardiovascular procedures, and receive cardiac transplants; there are few studies on this topic outside the US.^49,50,51,52,53^ There are many reasons to interpret existing data as evidence of surgical underuse for patients with lower income, particularly in the US, where employer-based insurance is common.^54,55,56,57^ At the same time, there is growing concern that at least part of wealth-based differences in utilization may represent overuse of certain treatments that are at best unnecessary and at worst harmful.^4,30,58,59,60,61^ If this is the case, residents of higher-income neighborhoods may be at increased risk of unnecessary or harmful care.

Third, our finding that the magnitude of the income-utilization gradient was generally larger in New York State and New South Wales than in Ontario is noteworthy. It begets important questions about potential overuse in the US and Australia, particularly among patients with higher incomes, as well as potential underuse in Canada.^62,63^ It is crucial to contemplate the role of health system organization and financing. Residents of Canada are served exclusively by a publicly funded single-payer health care system, while US residents and residents of Australia are served by a public-private hybrid.^23,64^ A recent study by Landon et al^65^ described Australia’s public-private hybrid delivery system. There is good reason to believe that single-payer systems, such as Canada, reduce income-based disparities and enhance equity, while public-private systems improve access problems inherent to single-payer models but potentiate increased utilization and inequities.^66^ Our work is consistent with a rich body of research by Gorey et al,^67,68^ who have published several international comparisons demonstrating improved cancer outcomes for low-income residents of Canada relative to their low-income US counterparts.

Fourth, our study highlights the importance of international comparative health services research and the powerful analyses that can be performed when population-based patient-level data from multiple countries are analyzed in parallel. Many studies, including those from The Commonwealth Fund and OECD, rely on aggregate data.^69^ Such studies are vital, but they lack the granularity required for fulsome comparison of disparities, utilization, and outcomes for individual diseases.^14,22,70,71,72^

Lastly, it is important to briefly mention some of our secondary outcomes and subgroup analyses. Our finding that a higher proportion of New York hospitals performed each surgery with more low-volume hospitals, relative to Ontario and New South Wales, highlights a continued lack of surgical regionalization in the US despite decades of research highlighting the importance of adequate procedural volumes.^33,34^ However, the nonsignificant differences in postoperative mortality outcomes and readmission rates in our study brings forward another aspect of surgical care quality across jurisdictions for each surgery despite greater regionalization in Ontario and New South Wales.

### Limitations

Our analyses are subject to several limitations. First, our analyses were limited to 1 geographic region in each of our 3 countries and should be generalized more broadly with care. Future work might examine these intercountry findings against intracountry (ie, state/provincial) comparisons. Second, our reliance on hospital administrative data prevented us from being able to ascertain indications for surgery or appropriateness, although we chose 3 conditions for which the indication for surgery is predominantly cancer.^35,73,74^ Further analyses using clinical registries is warranted. Likewise, the lack of clinical details in the administrative data sets precludes us from determining whether the higher rates of surgery in residents of higher-income neighborhoods compared with those of lower-income neighborhoods represent underuse in residents of lower-income neighborhoods or overuse in residents of higher-income neighborhoods; given the implications of potentially unnecessary procedures in those with higher-income or inadequate access in those with lower-income, further study is needed. Third, our data sources precluded us from evaluating longer-term or patient-reported outcomes. Fourth, we lacked information about radical prostatectomy performed in the outpatient setting; utilization of nonsurgical alternatives, such as radiation or watchful waiting, can be explored in the future.^37,42,75^ Fifth, given our large sample size and multiple comparisons, readers should interpret our subgroup and secondary analyses with caution, recognizing the potential for type 1 error.

## Conclusions

This study found higher overall utilization and a wider high income–low income gradient in New York and New South Wales than in Ontario for pancreatectomy, radical prostatectomy, and nephrectomy. These findings suggest that private health coverage in conjunction with public insurance systems may exacerbate income-based disparities in surgical utilization. Our study provides important evidence of the trade-offs in access and equity intrinsic to the health care systems of New York State (US), Ontario (Canada), and New South Wales (Australia).

## Notes

Supplement. **eFigure 1.** Flowchart for Cohort Generation of Patients Hospitalized for Pancreatectomy, Radical Prostatectomy, or Nephrectomy in New York State, Ontario, and New South Wales **eFigure 2.** Standardized Per Capita Utilization of Pancreatectomy, Radical Prostatectomy, and Nephrectomy (per 100 000 Population per Year) by Decade of Age in New York State, Ontario, and New South Wales **eTable 1.** Comorbid Conditions of Patients Who Underwent Pancreatectomy, Radical Prostatectomy, and Nephrectomy in New York State, Ontario, and New South Wales **eTable 2.** Unadjusted Outcomes of Patients Who Underwent Pancreatectomy, Radical Prostatectomy, and Nephrectomy in New York State, Ontario, and New South Wales **eTable 3.** Risk-Standardized Outcomes of Patients Who Underwent Pancreatectomy, Radical Prostatectomy, or Nephrectomy in New York State, Ontario, and New South Wales **eAppendix.** Procedure Codes for Pancreatectomy, Radical Prostatectomy, and Nephrectomy in New York State, Ontario, and New South Wales **eReferences.**
