# Supplementary material for: Utilization Rates of Pancreatectomy, Radical Prostatectomy, and Nephrectomy in New York, Ontario, and New South Wales, 2011 to 2018
Source: JAMA Netw Open. 2021 Apr 19;4(4):e215477. doi: 10.1001/jamanetworkopen.2021.5477 (PMC8056282; doi:10.1001/jamanetworkopen.2021.5477)
Supplement: Supplement. — eFigure 1. Flowchart for Cohort Generation of Patients Hospitalized for Pancreatectomy, Radical Prostatectomy, or Nephrectomy in New York State, Ontario, and New South Wales eFigure 2. Standardized Per Capita Utilization of Pancreatectomy, Radical Prostatectomy, and Nephrectomy (per 100 000 Population per Year) by Decade of Age in New York State, Ontario, and New South Wales eTable 1. Comorbid Conditions of Patients Who Underwent Pancreatectomy, Radical Prostatectomy, and Nephrectomy in New York State, Ontario, and New South Wales eTable 2. Unadjusted Outcomes of Patients Who Underwent Pancreatectomy, Radical Prostatectomy, and Nephrectomy in New York State, Ontario, and New South Wales eTable 3. Risk-Standardized Outcomes of Patients Who Underwent Pancreatectomy, Radical Prostatectomy, or Nephrectomy in New York State, Ontario, and New South Wales eAppendix. Procedure Codes for Pancreatectomy, Radical Prostatectomy, and Nephrectomy in New York State, Ontario, and New South Wales eReferences. [file jamanetwopen-e215477-s001.pdf]

Supplemental Online Content

Pang HYM, Chalmers K, Landon B, et al. Utilization rates of pancreatectomy, radical prostatectomy, and nephrectomy in New York, Ontario, and New South Wales, 2011 to 2018. *JAMA Netw Open.* 2021;4(4):e215477. doi:10.1001/jamanetworkopen.2021.5477

eFigure 1. Flowchart for Cohort Generation of Patients Hospitalized for Pancreatectomy, Radical Prostatectomy, or Nephrectomy in New York State, Ontario, and New South Wales

eFigure 2. Standardized Per Capita Utilization of Pancreatectomy, Radical Prostatectomy, and Nephrectomy (per 100 000 Population per Year) by Decade of Age in New York State, Ontario, and New South Wales

eTable 1. Comorbid Conditions of Patients Who Underwent Pancreatectomy, Radical Prostatectomy, and Nephrectomy in New York State, Ontario, and New South Wales

eTable 2. Unadjusted Outcomes of Patients Who Underwent Pancreatectomy, Radical Prostatectomy, and Nephrectomy in New York, Ontario, and New South Wales

eTable 3. Risk-Standardized Outcomes of Patients Who Underwent Pancreatectomy, Radical Prostatectomy, or Nephrectomy in New York State, Ontario, and New South Wales

eAppendix. Procedure Codes for Pancreatectomy, Radical Prostatectomy, and Nephrectomy in New York State, Ontario, and New South Wales

eReferences.

This supplemental material has been provided by the authors to give readers additional information about their work.

© 2021 Pang H et al. *JAMA Network Open.*

eFigure 1. Flowchart for Cohort Generation of Patients Hospitalized for Pancreatectomy, Radical Prostatectomy, or Nephrectomy in New York State, Ontario, and New South Wales*[*]

Records of hospitalizations with pancreatectomy, radical prostatectomy, or nephrectomy, as the primary procedure with a procedure date between January 1, 2011 (Ontario and New York) or January 1, 2013 (New South Wales) and March 31, 2018 (Ontario and New South Wales) or September 30, 2016 (New York) – no. of procedures (no. of patients)

	New York	Ontario	New South Wales
**Pancreatectomy**	7,074 (7,033)	5067 (5039)	2,192 (2,182)
**Radical prostatectomy**	26,102 (26,048)	19,173 (19,171)	14,057 (14,057)
**Nephrectomy**	29,646 (29,012)	17,612 (17,310)	7,315 (7,209)

EXCLUDED – no. of procedures

Hospitalization characteristics

- Discharge date outside study period or duplicated procedure within study period
- Duplicated procedure during 90-day look-back period
- Procedure not linked to a hospitalization

	New York	Ontario	New South Wales
**Pancreatectomy**	84	SC	22
**Radical prostatectomy**	126	0	12
**Nephrectomy**	273	44	47

Patient characteristics

- Age < 18 or > 105
- Missing age or sex (or female sex for radical prostatectomy)
- Residential postal code outside of jurisdiction
- Length of stay < 1 day
- Death on or prior to procedure date

	New York	Ontario	New South Wales
**Pancreatectomy**	1176	51	67
**Radical prostatectomy**	4140	14	543
**Nephrectomy**	4298	385	389

Hospital characteristics

- Records of procedures performed in hospitals with mean volume <1 per year

	New York	Ontario	New South Wales
**Pancreatectomy**	73	60	25
**Radical prostatectomy**	65	32	SC
**Nephrectomy**	75	20	SC

* SC = Cell sizes n $\leq$ 5 (Ontario and New South Wales) or n $\leq$ 10 (New York) and suppressed in accordance with jurisdictional research ethics board policy

© 2021 Pang H et al. *JAMA Network Open.*

Final study cohort – no. of procedures (no. of patients)

	New York	Ontario	New South Wales
**Pancreatectomy**	5,741 (5,717)	4,953 (4,929)	2,078 (2,069)
**Radical prostatectomy**	21,771 (21,752)	19,127 (19,125)	13,499 (13,499)
**Nephrectomy**	25,000 (24,617)	17,163 (16,916)	6,874 (6,804)

eFigure 2. Standardized Per Capita Utilization of Pancreatectomy, Radical Prostatectomy, and Nephrectomy (per 100 000 Population per Year) by Decade of Age in New York State, Ontario, and New South Wales.

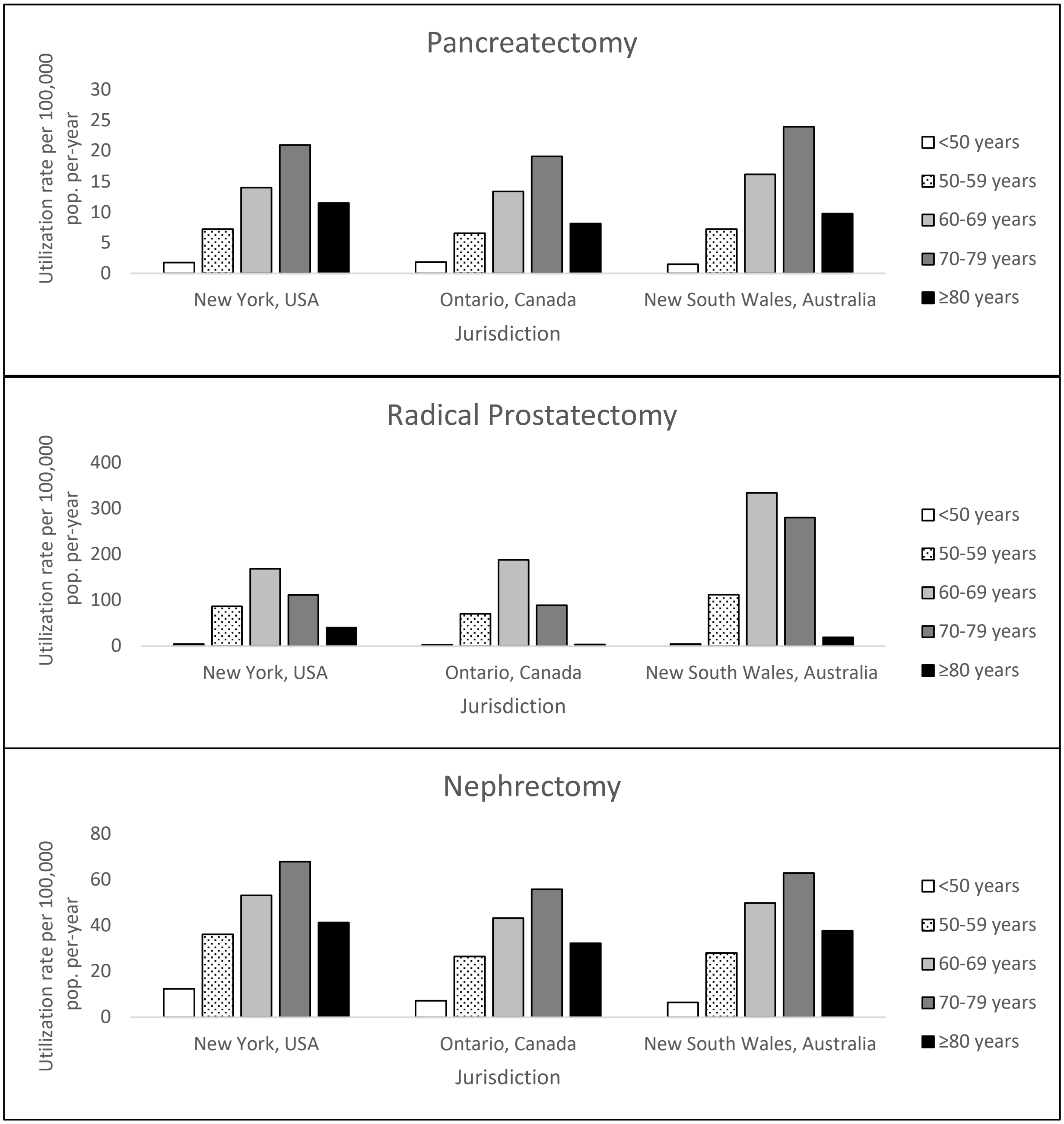

eTable 1. Comorbid Conditions of Patients Who Underwent Pancreatectomy, Radical Prostatectomy, and Nephrectomy in New York State,Ontario, and New South Wales

	Pancreatectomy				Radical Prostatectomy				Nephrectomy			
	New York (N=5,717)	Ontario (N= 4,929)	New South Wales (N=2,069)	P-value	New York (N=21,752)	Ontario (N=19,125)	New South Wales (N=13,499)	P-value	New York (N=24,617)	Ontario (N=16,916)	New South Wales (N=6,804)	P-value
**Comorbid conditions – no. (%)**												
Congestive heart failure	202 (3.5)	62 (1.3)	40 (1.9)	<.001	396 (1.8)	42 (0.2)	23 (0.17)	<.001	989 (4.0)	248 (1.5)	52 (0.8)	<.001
Depression	476 (8.3)	95 (1.9)	28 (1.4)	<.001	1050 (4.8)	59 (0.3)	13 (0.10)	<.001	1828 (7.4%)	110 (0.7)	19 (0.3)	<.001
Hypertension with complications	227 (4.0)	SC †	SC	<.001	621 (2.9)	9 (0.0)	SC	<.001	2717 (11.0)	144 (0.9)	53 (0.8)	<.001
Diabetes without complications	1433 (25.1)	783 (15.9)	283 (13.7)	<.001	3053 (14.0)	1432 (7.5)	1246 (9.2)	<.001	4366 (17.7)	1780 (10.5)	660 (9.7)	<.001
COPD	834 (14.6)	194 (3.9)	38 (1.8)	<.001	1815 (8.3)	348 (1.8)	53 (0.4)	<.001	3432 (13.9)	588 (3.5)	93 (1.4)	<.001
Renal Failure	244 (4.3)	73 (1.5)	59 (2.9)	<.001	700 (3.2)	70 (0.4)	87 (0.6)	<.001	3084 (12.5)	1108 (6.6)	676 (9.9)	<.001
An associated cancer diagnosis	2952 (51.6)	2505 (50.8)	1528 (73.9)	<.001	19128 (87.9)	18914 (98.9)	13433 (99.5)	<.001	13754 (55.9)	13262 (78.4)	5303 (77.9)	<.001

† SC = Cell sizes n ≤ 5 (Ontario and New South Wales) or n ≤ 10 (New York) and suppressed in accordance with jurisdictional research ethics board policy

© 2021 Pang H et al. *JAMA Network Open.*

eTable 2. Unadjusted Outcomes of Patients Who Underwent Pancreatectomy, Radical Prostatectomy, and Nephrectomy in New York, Ontario, and New South Wales.‡

	Pancreatectomy				Radical Prostatectomy				Nephrectomy			
	**New York** (N=5,717)	**Ontario** (N=4,929)	**New South Wales** (N=2,069)	**P-value**	**New York** (N=21,752)	**Ontario** (N=19,125)	**New South Wales** (N=13,499)	**P-value**	**New York** (N=24,617)	**Ontario** (N=16,916)	**New South Wales** (N=6,804)	**P-value**
**Hospital length of stay (LOS), days**												
Mean (SD)	11.5 (10.2)	13.3 (13.4)	17.8 (13.8)	<.001	2.2 (2.8)	2.7 (2.2)	4 (3.8)	<.001	4.4 (5.5)	5.7 (7.2)	6.4 (6.3)	<.001
Median (interquartile range)	8 (7.0)	9 (8.0)	13 (12.0)	NA‡	1 (1.0)	2 (1.0)	3 (2.0)	NA	3.0 (3.0)	4 (3.0)	5 (3.0)	NA
LOS > 100 days – no. (%)	11 (0.2)	62 (1.3)	15 (0.7)	<.001	SC	SC	SC	0.63	10 (0.0)	46 (0.3)	7 (0.1)	<.001
**Discharge disposition – no. (%)§**												
Death, in-hospital	91 (1.6)	145 (2.9)	46 (2.2)	<.001	13 (0.1)	9 (0.0)	9 (0.1)	0.74	93 (0.4)	154 (0.9)	14 (0.2)	<.001
Death within 7-days post-surgery	30 (0.5)	42 (0.8)	17 (0.8)	0.095	SC	SC	SC	0.41	36 (0.1)	56 (0.3)	6 (0.1)	<.001
Home	5126 (89.3)	4574 (92.3)	1866 (89.8)	<.001	21447 (98.5)	19082 (99.8)	13223 (98.0)	<.001	23819 (95.3)	16657 (97.1)	6587 (95.8)	<.001
Long-term care, nursing care, or rehabilitation centre	500 (8.7)	212 (4.3)	114 (5.5)	<.001	285 (1.3)	29 (0.2)	233 (1.7)	<.001	1014 (4.1)	318 (1.9)	176 (2.6)	<.001
Other	23 (0.4)	22 (0.4)	52 (2.5)	<.001	26 (0.1)	7 (0.0)	30 (0.2)	<.001	74 (0.3)	34 (0.2)	96 (1.4)	<.001
**Readmission – no. (%)**												
30-day readmission	1207 (21.4)	876 (18.2)	351 (17.3)	<.001	1106 (5.1)	856 (4.5)	803 (6.0)	<.001	2130 (8.6)	1230 (7.2)	485 (7.1)	<.001
90-day readmission	1654 (29.3)	1262 (26.2)	530 (26.1)	0.001	1600 (7.4)	1163 (6.1)	1095 (8.1)	<.001	3513 (14.1)	2024 (11.9)	799 (11.6)	<.001

‡ Not applicable (NA)
§ Discharge disposition % may add up to more than 100% since died within 7-days of surgery and died in-hospital overlap with each other

© 2021 Pang H et al. *JAMA Network Open.*

eTable 3. Risk-Standardized Outcomes of Patients Who Underwent Pancreatectomy, Radical Prostatectomy, or Nephrectomy in New York State, Ontario, and New South Wales

	Pancreatectomy							
	Model 1**				Model 2††			
	**New York**	**Ontario**	**New South Wales**	**P-value**	**New York**	**Ontario**	**New South Wales**	**P-value**
Hospital LOS, days – mean (95% CI)	12.0 (11.6, 12.4)	14.4 (13.8, 15.0)	18.4 (17.5, 19.3)	<.001	13.3 (12.5, 14.2)	15.8 (14.7, 16.8)	19.4 (17.8, 21.0)	<.001
**Discharge disposition - % (95% CI)**								
Death, in-hospital	1.7 (1.2, 2.3)	3.7 (3.0, 4.5)	2.5 (1.6, 3.7)	<.001	2.2 (1.2, 3.9)	3.6 (2.4, 5.4)	2.8 (1.4, 5.3)	<.001
Death within 7-days of surgery	0.4 (0.2, 0.8)	1.1 (0.8, 1.6)	0.8 (0.4, 1.6)	<.001	0.8 (0.3, 1.8)	1.3 (0.6, 2.7)	1.7 (0.7, 4.2)	0.002
Home	94.0 (93.0, 94.9)	92.3 (91.1, 93.4)	93.9 (92, 95.3)	0.001	91.0 (88.6, 93)	90.4 (87.7, 92.6)	93.9 (91, 95.9)	<.001
Long-term care, nursing care, or rehabilitation centre	3.9 (3.2, 4.7)	3.2 (2.5, 4.0)	2.2 (1.5, 3.3)	0.001	6.1 (4.6, 8.0)	4.6 (3.1, 6.6)	2.0 (1.0, 3.8)	<.001
Other	0.4 (0.2, 0.8)	0.6 (0.4, 1.0)	1.5 (0.9, 2.6)	<.001	0.4 (0.1, 1.4)	0.9 (0.3, 2.5)	1.6 (0.8, 3.0)	<.001
**Readmission - % (95% CI)**								
30-day readmission	22.7 (21.2, 24.3)	20.4 (18.9, 22.0)	18.8 (16.5, 21.4)	<.001	22.9 (19.9, 26.3)	19.4 (16.5, 22.7)	22 (17.4, 27.3)	<.001
90-day readmission	30.2 (28.5, 32.0)	28.3 (26.6, 30.2)	28.8 (26.1, 31.7)	0.096	30.7 (27.4, 34.3)	28.4 (25.0, 32.0)	31.6 (26.5, 37.3)	0.007
	Radical Prostatectomy							
	Model 1				Model 2			
	**New York**	**Ontario**	**New South Wales**	**P-value**	**New York**	**Ontario**	**New South Wales**	**P-value**
Hospital LOS, days – mean (95% CI)	2.2 (2.1, 2.2)	2.7 (2.6, 2.7)	3.8 (3.8, 3.9)	<.001	2.5 (2.4, 2.6)	3.0 (2.9, 3.1)	4.6 (4.4, 4.7)	<.001
**Discharge disposition - % (95% CI)**								
Death, in-hospital	0.02 (0.01, 0.05)	0.01 (0.00, 0.03)	0.0 (0.0, 0.1)	0.50	0.04 (0.02, 0.10)	-	0.0 (0.0, 0.2)	NA
Death within 7-days of surgery	0.02 (0.01, 0.04)	0.01 (0.00, 0.06)	0.0 (0.0, 0.1)	0.50	0.02 (0.00, 0.09)	-	-	NA
Home	99.3 (99.2, 99.4)	99.8 (99.7, 99.9)	98.1 (97.8, 98.3)	<.001	99.2 (98.8, 99.4)	99.7 (99.4, 99.8)	94.6 (93.5, 95.6)	<.001
Long-term care, nursing care, or rehabilitation centre	0.5 (0.4, 0.6)	0.1 (0.1, 0.2)	1.7 (1.5, 2.0)	<.001	0.6 (0.4, 0.9)	0.2 (0.1, 0.4)	5.1 (4.2, 6.2)	<.001
Other	0.1 (0.1, 0.2)	0.03 (0.02, 0.08)	0.1 (0.1, 0.2)	0.005	0.1 (0, 0.4)	-	0.2 (0.1, 0.5)	NA
**Readmission - % (95% CI)**								
30-day readmission	4.9 (4.6, 5.2)	4.5 (4.2, 4.8)	5.9 (5.5, 6.3)	<.001	5.5 (4.7, 6.4)	3.9 (3.3, 4.5)	7.2 (6.2, 8.5)	<.001
90-day readmission	7.0 (6.6, 7.3)	6.0 (5.7, 6.4)	8 (7.5, 8.5)	<.001	7.7 (6.8, 8.7)	5.8 (5.0, 6.6)	8.8 (7.7, 10.1)	<.001
	Nephrectomy							
	Model 1				Model 2			
	**New York**	**Ontario**	**New South Wales**	**P-value**	**New York**	**Ontario**	**New South Wales**	**P-value**

** Model 1: adjusted for patient age and sex
†† Model 2: adjusted for Model 1 plus neighborhood income and hospital volume

© 2021 Pang H et al. *JAMA Network Open.*

Hospital LOS, days – mean (95% CI)	4.6 (4.5, 4.7)	5.6 (5.5, 5.8)	6.4 (6.2, 6.6)	<.001	4.9 (4.7, 5.0)	5.4 (5.1, 5.6)	6.6 (6.2, 7)	<.001
**Discharge disposition - % (95% CI)**								
Death, in-hospital	0.3 (0.2, 0.4)	0.7 (0.5, 0.9)	0.2 (0.1, 0.4)	<.001	0.4 (0.2, 0.6)	0.5 (0.3, 0.8)	0.2 (0.1, 0.8)	0.002
Died within 7-days of surgery	0.1 (0.1, 0.2)	0.3 (0.2, 0.4)	0.1 (0.0, 0.3)	<.001	0.2 (0.1, 0.4)	0.2 (0.1, 0.5)	0.2 (0.1, 0.7)	0.97
Home	96.8 (96.4, 97.1)	97.9 (97.6, 98.2)	97.6 (97.0, 98.1)	<.001	95.7 (94.9, 96.3)	98.0 (97.5, 98.4)	97.3 (96.3, 98.0)	<.001
Transfer to long-term care, nursing care, or rehabilitation centre	2.5 (2.2, 2.8)	1.1 (0.9, 1.4)	1.6 (1.2, 2.1)	<.001	3.3 (2.8, 4.0)	1.2 (0.9, 1.7)	1.9 (1.3, 2.8)	<.001
Other	0.3 (0.3, 0.5)	0.3 (0.2, 0.4)	0.5 (0.3, 0.8)	0.96	0.5 (0.3, 0.9)	0.2 (0.1, 0.5)	0.5 (0.3, 1.0)	<.001
**Readmission - %, (95% CI)**								
30-day readmission	9.0 (8.5, 9.5)	7.3 (6.8, 7.8)	7.2 (6.5, 8.0)	<.001	8.4 (7.5, 9.4)	6.6 (5.7, 7.5)	7.4 (6.1, 8.9)	<.001
90-day readmission	15.0 (14.4, 15.6)	11.9 (11.3, 12.6)	11.9 (10.9, 12.9)	<.001	14.8 (13.6, 16.1)	10.9 (9.8, 12.1)	12.3 (10.6, 14.2)	<.001

© 2021 Pang H et al. *JAMA Network Open.*

eAppendix. Procedure Codes for Pancreatectomy, Radical Prostatectomy, and Nephrectomy in New York State, Ontario, and New South Wales

Pancreatectomy

New York State, USA

ICD-9[1]	Description
52.5	Partial pancreatectomy
52.51	Proximal pancreatectomy with synchronous duodenectomy
52.52	Distal pancreatectomy
52.53	Radical subtotal pancreatectomy
52.59	Other partial pancreatectomy
52.6	Total pancreatectomy<br>Includes: Pancreatectomy with synchronous duodenectomy
52.7	Radical pancreatectomy

ICD-10[2]	Description
0FTG0ZZ	Resection of Pancreas, Open Approach
0FTG4ZZ	Resection of Pancreas, Percutaneous Endoscopic Approach
0FBG0ZZ	Excision of Pancreas, Open Approach
0FBG3ZZ	Excision of Pancreas, Percutaneous Approach
0FBG4ZZ	Excision of Pancreas, Percutaneous Endoscopic Approach

Ontario, Canada

CCI[3]	Description
1.OJ.87.^^	Excision partial, pancreas
1.OJ.89.^^	Excision total, pancreas
1.OK.87.^^	Excision partial, pancreas with duodenum
1.OK.89.^^	Excision total, pancreas with duodenum
1.OK.91.^^	Excision radical, pancreas with duodenum

New South Wales, Australia

ACHI[4]	Description
30584-00	Pancreaticoduodenectomy with formation of stoma
30593-00	Pancreatectomy
30583-00	Distal pancreatectomy
30593-01	Pancreatectomy with splenectomy

© 2021 Pang H et al. *JAMA Network Open.*

Radical Prostatectomy

New York State, USA

ICD-9[5–7]	Description
60.4	Retropubic prostatectomy
60.5	Radical prostatectomy
60.69	Other prostatectomy
54.21<br>54.51	Radical prostatectomy, laparoscopic
17.4^	Radical prostatectomy, robotic
**ICD10[8]**	**Description**
0VT0^^	Resection of the prostate
0VB0^^	Excision of the prostate<br>0VB03ZZ<br>0VB00ZZ<br>0VB04Z
0V50^^	Destruction of the prostate
0VP4^^	Removal of the prostate
8E0W^^	Robotic procedure included when listed as the primary procedure code when in conjunction with a prostate specific code (above) as a secondary code

Ontario, Canada

CCI[9]	Description
1.QT.91.^^	Excision radical, prostate
1.QT.91.PB	Excision radical, prostate—Open Surgery (perineal approach)
1.QT.91.PK	Excision radical, prostate—Open Surgery (retropubic approach)
1.QT.91.DA	Excision radical, prostate—Laparoscopic Surgery
1.QT.91.BQ	Excision radical, prostate—Robotic Assistance (2009 to 2011)
7.SF.14	Excision radical, prostate—Robotic Assistance (2012)

New South Wales, Australia

ACHI[4]	Description
37209-01	Laparoscopic radical prostatectomy
37210-01	Laparoscopic radical prostatectomy with bladder neck reconstruction
37211-01	Laparoscopic radical prostatectomy with bladder neck reconstruction and pelvic lymphadenectomy
37209-00	Radical prostatectomy
37210-00	Radical prostatectomy with bladder neck reconstruction
37211-00	Radical prostatectomy with bladder neck reconstruction and pelvic lymphadenectomy

© 2021 Pang H et al. *JAMA Network Open.*

Nephrectomy

New York State, USA

ICD-9[10–12]	Description
55.4	Partial nephrectomy
55.50	Complete nephrectomy
55.51	Nephroureterectomy
55.52	Nephrectomy of remaining kidney
55.54	Bilateral nephrectomy
55.53	Removal of transplanted kidney

ICD-10[13]	Description
0TT10ZZ	Resection of Left Kidney, Open Approach
0TT14ZZ	Resection of Left Kidney, Percutaneous Endoscopic Approach
0TT00ZZ	Resection of Right Kidney, Open Approach
0TT04ZZ	Resection of Right Kidney, Percutaneous Endoscopic Approach
0TT40ZZ	Resection of Left Kidney Pelvis, Open Approach
0TT44ZZ	Resection of Left Kidney Pelvis, Percutaneous Endoscopic
0TT47ZZ	Resection of Left Kidney Pelvis, Via Natural or Artificial Opening
0TT48ZZ	Resection of Left Kidney Pelvis, Via Natural or Artificial Opening
0TT30ZZ	Resection of Right Kidney Pelvis, Open Approach
0TT34ZZ	Resection of Right Kidney Pelvis, Percutaneous Endoscopic Approach
0TT37ZZ	Resection of Right Kidney Pelvis, Via Natural or Artificial Opening
0TT38ZZ	Resection of Right Kidney Pelvis, Via Natural or Artificial Opening

Ontario, Canada

CCI[3,14]	Description
1.PC.87.^^	Excision partial, kidney
1.PC.87.LA	open approach using apposition technique [e.g. suturing, stapling]
1.PC.87.LA-XX-E	open approach using local flap closure
1.PC.87.LA-XX-G	open approach using pedicled flap
1.PC.87.DA	endoscopic [laparoscopic, laparoscopicassisted, hand-assisted] approach using apposition technique [e.g. suturing, stapling]
1.PC.87.NQ	open bench surgery approach using apposition technique [e.g. suturing, stapling]
1.PC.89.^^	Excision total, kidney
1.PC.89.DA	using endoscopic (laparoscopic, laparoscopic-assisted, hand-assisted) approach
1.PC.89.LB	using open abdominal approach
1.PC.89.PF	using open lumbar [flank] approach
1.PC.89.QF	using open thoracoabdominal approach
1.PC.91.^^	Excision radical, kidney
1.PC.91.DA	using endoscopic [laparoscopic, laparoscopic-assisted, hand-assisted] approach
1.PC.91.LB	using open abdominal approach
1.PC.91.PF	using open lumbar [flank] approach
1.PC.91.QF	using open thoracoabdominal approach

New South Wales, Australia

ACHI[4]	Description
36522-00	Laparoscopic partial nephrectomy
36522-02	Other closed partial nephrectomy; Percutaneous resection of lesion of kidney via nephrostomy
36522-01	Partial nephrectomy

© 2021 Pang H et al. *JAMA Network Open.*

36525-00	Laparoscopic partial nephrectomy complicated by previous surgery on same kidney
36525-02	Other closed partial nephrectomy complicated by previous surgery on same kidney
36525-01	Partial nephrectomy complicated by previous surgery on same kidney
36516-00	Laparoscopic complete nephrectomy, unilateral; Removal of remaining (solitary) kidney, via laparoscopy
36516-01	Complete nephrectomy, unilateral; Removal of remaining (solitary) kidney
36516-02	Laparoscopic complete nephrectomy, bilateral
36516-03	Complete nephrectomy, bilateral
36528-00	Laparoscopic radical nephrectomy
36528-01	Radical nephrectomy
36529-00	Radical nephrectomy complicated by previous surgery on same kidney
36531-01	Nephroureterectomy; Nephrectomy with total ureterectomy
36533-00	Nephroureterectomy complicated by previous surgery on same kidney; Nephrectomy with total ureterectomy complicated by previous surgery on the same kidney
36531-00	Laparoscopic nephroureterectomy; Nephrectomy with total ureterectomy via laparoscopy
36519-01	Laparoscopic complete nephrectomy for removal of transplanted kidney
36519-02	Complete nephrectomy for removal of transplanted kidney

© 2021 Pang H et al. *JAMA Network Open.*

eReferences

1 Amini N, Kim Y, Hyder O, Spolverato G, WU C, Page A, et al. A nationwide analysis of the use and outcomes of perioperative epidural analgesia in patients undergoing hepatic and pancreatic surgery. *Am J Surg* 2015;210(3):483–91.

2 Agency for Healthcare Research and Quality. AHRQ Quality Indicators™ (AHRQ QI™) ICD-9-CM and ICD-10-CM/PCS Specification Enhanced Version 5.0: Inpatient Quality Indicators #2 (IQI #2) Pancreatic Resection Volume. Rockville, MD; United States Department of Health and Human Services; 2015.

3 McIsaac DI, Wijeysundera DN, Huang A, Bryson GL, van Walraven C. Association of the hospital volume of frail surgical patients cared for with outcomes after elective, major noncardiac surgery. *Anesthesiology* 2017;126(4):602–13.

4 Australian Consortium for Classification Development (ACCD). Australian Classification of Health Interventions (ACHI) 10th edition. 2016.

5 Trinh Q-D, Sammon J, Sun M, Ravi R, Ghani K, Bianchi M, et al. Perioperative outcomes of robot-assisted radical prostatectomy compared with open radical prostatectomy: Results from the nationwide inpatient sample. *Eur Urol* 2011;61(4):679–85.

6 Savage CJ, Vickers A. Low annual caseloads in US Surgeons conducting radical prostatectomy. *J Urol* 2009;182(6):2677–9.

7 Tyson MD, Andrews PE, Ferrigni RF, Humphreys MR, Parker AS, Castle EP. Radical prostatectomy trends in the United States: 1998 to 2011. *Mayo Clin Proc* 2016;91(1):10–6.

8 Boston Scientific. Prostate Health: 2018 Coding & Payment Quick Reference. Marlborough, MA; Boston Scientific Corporation; 2018.

9 Canadian Institute for Health Information. The delivery of radical prostatectomy to treat men with prostate cancer. Ottawa, ON; Canadian Insititute for Health Information; 2014.

10 Joudi FN, Allareddy V, Kane CJ, Konety BR. Analysis of complications following partial and total nephrectomy for renal cancer in a population based sample. *J Urol* 2007;177(5):1709–14.

11 Bjurlin M, Walter D, Taksler G, Huang W, Wysock J, Sivarajan G, et al. National trends in the utilization of partial nephrectomy before and after the establishment of AUA guidelines for the management of renal masses. *Urology* 2013;82(6):1–11.

12 Liss MA, Wang S, Palazzi K, Jabaji R, Patel N, Lee HJ, et al. Evaluation of national trends in the utilization of partial nephrectomy in relation to the publication of the American Urologic Association guidelines for the management of clinical T1 renal masses. *BMC Urol* 2014;14(1):1–10.

13 Centers for Medicare and Medicaid Services. 2018 ICD-10 CM and general equivalence mapping. Centres for Medicare and Medicaid Services; 2018.

14 Abouassaly R, Alibhai SMH, Tomlinson G, Timilshina N, Finelli A. Unintended consequences of laparoscopic surgery on partial nephrectomy for kidney cancer. *J Urol* 2010;183(2):467–72.

© 2021 Pang H et al. *JAMA Network Open.*
